# Viable but Nonculturable Bacteria: Food Safety and Public Health Perspective

**DOI:** 10.1155/2013/703813

**Published:** 2013-09-26

**Authors:** Md. Fakruddin, Khanjada Shahnewaj Bin Mannan, Stewart Andrews

**Affiliations:** ^1^Industrial Microbiology Laboratory, Institute of Food Science and Technology (IFST), Bangladesh Council of Scientific and Industrial Research (BCSIR), Dhaka, Bangladesh; ^2^Center for Food and Waterborne Diseases, icddr, b, Dhaka, Bangladesh; ^3^Oakden-Osmosis, Australia

## Abstract

The viable but nonculturable (VBNC) state is a unique survival strategy of many bacteria in the environment in response to adverse environmental conditions. VBNC bacteria cannot be cultured on routine microbiological media, but they remain viable and retain virulence. The VBNC bacteria can be resuscitated when provided with appropriate conditions. A good number of bacteria including many human pathogens have been reported to enter the VBNC state. Though there have been disputes on the existence of VBNC in the past, extensive molecular studies have resolved most of them, and VBNC has been accepted as a distinct survival state. VBNC pathogenic bacteria are considered a threat to public health and food safety due to their nondetectability through conventional food and water testing methods. A number of disease outbreaks have been reported where VBNC bacteria have been implicated as the causative agent. Further molecular and combinatorial research is needed to tackle the threat posed by VBNC bacteria with regard to public health and food safety.

## 1. Introduction

The cells that form a colony on specific nutrient media are the culturable cells. Viable means metabolically or physiologically active. So the cells that are metabolically or physiologically active but cannot be cultured on specific media are the viable but nonculturable cells (VBNC) [1]. Most microorganisms growing in nature have yet to be cultured in the laboratory. In fact, less than 1% of the microorganisms in natural water and soil samples are cultured in viable count procedures [2].

In 1982, Xu and coworkers introduced the term “viable but nonculturable bacterial cells (VBNC)” to distinguish particular cells that could not form colonies on solid media but retained metabolic activity and the ability to elongate after the administration of nutrients [3]. According to Oliver [4], “VBNC can be defined as a metabolically active bacterial cell that crossed a threshold in this way, for known or unknown reasons, and became unable to multiply in or on a medium normally supporting its growth.” Most of the bacteria that enter VBNC state are gram-negative species belonging to the gamma subclass of the proteobacteria branch, except for *Rhizobium*, *Agrobacterium,* and *Helicobacter-Campylobacter* species [5].

## 2. History

Bashford and colleagues [6] announced that they had recovered *Vibrio cholerae* from streams and drainage ditches, including sites with negligible chance of sewage contamination. Around the same time, Colwell et al. [7] also found *Vibrio cholerae* in Maryland, USA. She and her coworkers showed that, in artificial sea water, *Vibrio cholera *and *E. coli *remained viable though they lost the capacity to form colonies on culture media [8]. Soon *Salmonella enteritidis*, *Shigella sonnei,* and *Legionella pneumophila* joined the list of organisms known to be capable of entering a state in which they failed to show up on nutrient agar yet took up substrates and signaled in other ways that they were certainly not dead [9]. The use of laboratory media to recover and enumerate bacteria and to link them with or absolve them from pathological and other activities became obsolete by the new discoveries, and the term VBNC (viable but nonculturable) was introduced [10].

## 3. VBNC

Microorganisms that do not grow in culture media, but are still metabolically active and capable of causing infections in animals and plants, are said to be in a VBNC state. Traditional laboratory culture conditions and methods cannot meet the requirements of VBNC organisms to resume growth [11]. Semistarved bacteria usually resume growth immediately when appropriate nutrients and conditions are provided. Viable but nonculturable cells will not resume growth even when nutrients are provided [12]. VBNC cells exhibit active metabolism in the form of respiration or fermentation, incorporate radioactive substrates, and have active protein synthesis but cannot be cultured or grown on conventional laboratory media. They have been detected by observing discrepancies between plate count enumeration of bacterial population and direct staining and microscopic counts [13]. These cells may be a problem in the environment if they are pathogens. For example, viable but nonculturable cells of *Vibrio cholerae, *enteropathogenic *E. coli*, *Legionella pneumophila,* and various other bacteria have been shown to regain culturability after they have entered the intestinal tracts of animals [14].

The VBNC state is defined as a state of dormancy triggered by harsh environmental conditions [15], such as nutrient starvation [16], extreme temperatures [17], and sharp changes in pH or salinity [18]; osmotic stress [19], oxygen availability [20, 21], and damage to or lack of an essential cellular component including DNA; exposure to food preservatives [22] and heavy metals [23, 24]; exposure to white light [25]; activation of lysogenic phages or suicide genes such as *sok/hak* or autolysins [26]; and decontaminating processes such as pasteurization of milk [27] and chlorination of wastewater [28].

The characteristics of bacteria in the VBNC state can be summarized as follows:

- maintaining apparent cell integrity;
- possessing some form of measurable cellular activity [29];
- possessing apparent capacity to regain culturability *in vivo* [30];
- responding to external stimulus as shown by specific gene expression [31];
- having low metabolic activity [28];
- exhibiting dwarfing [32];
- having reduced nutrient transport;
- containing a high ATP level and exhibit high membrane potential [33];
- having extensive modifications to the fatty acid composition in cytoplasmic membranes [34];
- within the cell wall peptidoglycan, increased crosslinking, increased muropeptides bearing covalently bound lipoprotein, and shortening of the average length of glycan strands [35];
- higher autolytic capability than exponentially growing cells;
- retained plasmids;
- increased antibiotic resistance due to lower metabolic activity [15];
- changes in outer-membrane protein profile [36];
- continuous gene expression [37].

The VBNC state continues to be disputed due to the difficulties of differentiating VBNC cells and dormant cells through resuscitation and phenotypic studies. However, recent molecular studies have generated data to support the existence of the VBNC state [38].

## 4. VBNC Pathogens

The VBNC is also considered an important reservoir of many human pathogens in the environment [39].

The following list includes but is not limited to the pathogenic bacteria that can enter the VBNC state [15]: *Aeromonas hydrophila, Agrobacterium tumefaciens, Burkholderia cepacia, Campylobacter jejuni, Enterobacter aerogenes, Enterobacter cloacae, Enterococcus faecalis, Escherichia coli (including EHEC), Helicobacter pylori, Klebsiella pneumoniae, Legionella pneumophila, Listeria monocytogenes, Mycobacterium tuberculosis, Pseudomonas aeruginosa, Salmonella typhi, Salmonella typhimurium, Shigella dysenteriae, Shigella flexneri, Shigella sonnei, Streptococcus faecalis, Vibrio alginolyticus, Vibrio cholerae, Vibrio harveyi, Vibrio parahaemolyticus, and Vibrio vulnificus (types 1 and 2).*

## 5. Public Health Significance of VBNC

Many believe that pathogens in the VBNC state are unable to induce infection/disease despite retaining their virulent properties. However, when VBNC pathogens pass through a host animal [40], resuscitation and resumption of metabolic activity have led to infections and diseases [40, 41]. The first evidence of pathogenicity of nonculturable cells was the demonstration of fluid accumulation in the rabbit ileal loop assay (RICA) by VBNC *Vibrio cholerae* O1, followed by human volunteer experiments [42]. VBNC *E. coli* nonculturable cells were also reisolated after passaging through rabbit ileal loops 4 days after inoculation, and chick embryos died when injected with nonculturable cells of *Legionella pneumophila*, leading to the conclusion that VBNC pathogens remain potentially pathogenic. So, VBNC has a huge significance in public health care [43].

Many indicator bacteria and pathogenic bacteria which exist in aquatic habitats have been shown to have a VBNC state [38]. Water is routinely tested for such indicators and pathogens, and if they are not detected or enumerated at a concentration below guidelines, waters are deemed to be safe for public consumption [44]. Therefore, where circumstances indicate possible presence of VBNC pathogens, additional molecular methodology needs to be used to reduce the risk of infective disease outbreaks [45].

Thus, food and environmental and clinical samples no longer can be considered free from pathogens if culturing yields negative results. For the general public, the presence of VBNC in water and food may be related to low-grade infections or the so-called aseptic infections. In many cases, the infections are incorrectly attributed to viruses since no bacteria were detected. For example, *Vibrio cholerae* O1 in the surface water have been shown to remain in nonculturable state. These water sources are used for domestic purpose regularly and pose a risk of infection [46]. When conditions are not favorable for growth, then it transforms to the nonculturable state in association with crustacean copepods. Persistence of *Vibrio cholerae* in water in the VBNC state is an important public health factor, since detection will not be successful if only conventional cultural methods are used [47].

Similarly, *Shigella* can undergo VBNC state in water but become a threat when they enter the human body [48]. Additional studies have indicated that a good number of pathogenic bacteria can survive food and water treatment processes and persist as well as retain virulence in processed food, pasteurized milk, potable water, and the environment [49]. One study demonstrated that recurrent urinary tract infections in many individuals were caused by uropathogenic *E. coli* cells which remained in VBNC state [50]. Furthermore, these VBNC cells demonstrated resistance to antibiotic treatment causing reinfection [51]. Other studies showed that uropathogenic VBNC *E. coli* retain enteropathogenicity as shown by continual production of enterotoxin [52]. Nilsson et al. [53] showed that VBNC *Helicobacter pylori* cells can express virulence factors such as *cagA*, *vacA,* and *vreA*.

## 6. VBNC State of Foodborne Bacteria: A Challenge in Food Safety

The presence of VBNC bacteria in food is well documented [54]. Food and its surrounding environment are a complex system, in which physiochemical characteristics (pH, *a*_*w*_, and chemical composition) and environmental factors (storage temperature and time, decontamination treatments, and packaging under modified atmosphere) act simultaneously on contaminating bacteria leading to the VNBC state [55]. For example, it has been demonstrated that refrigerated pasteurized grapefruit juice induced the VBNC state in *E. coli* O157 : H7 and *S. typhimurium* within 24 hours of incubation [56]. Again, Gunasekera et al. [27] reported that in pasteurized milk which has undergone thermal treatment, contaminating bacteria such as *E. coli* and *Pseudomonas putida* enter into the VBNC state but retain transcription and translation functions. Several foodborne outbreaks have been reported in Japan, where pathogens such as *Salmonella enterica* subsp. *enterica* [57] and *E. coli* O157 [58] in the VBNC state were responsible for outbreaks.

The VNBC is also critical in determining shelf life and microbial stability of food and beverages. For example, acetic acid and lactic acid bacteria entered the VBNC state in wine as a consequence of lack of oxygen and presence of sulphites [59].

## 7. Methods of Detection of VBNC Bacteria

### 7.1. Bright Field Microscopy with Nalidixic Acid

Nalidixic acid (20–40 mg/L) is used to stop cell division. After exposure to nalidixic acid, viable cells continue to grow and will appear elongated, whereas the nonviable metabolically inactive cells will retain their original shape and size. The cells are then observed under a microscope. Viable cells will be seen as elongated, whereas VBNC/dormant cells will be seen as oval and large [60].

### 7.2. Fluorescent Microscopy

Various fluorescent staining procedures can be used to determine VBNC organisms. Frequently used stains are acridine orange, 4,6-diamino-2-phenyl indole (DAPI), fluorescein isothiocyanate (FITC), indophenyl-nitrophenyl-phenyltetrazolium chloride (INT), and 5-cyano-2,3-ditolyl tetrazolium chloride (CTC) [61]. The mode of action of these dyes and the reactions observed are summarised in Table 1.

In recent years, a new differential staining assay, the *BacLight* Live/Dead assay, has been developed. The assay allows simultaneous counting total and viable (metabolically active) cells, by using two nucleic acid stains, that is, green-fluorescent SYTO 9 stain and red-fluorescent propidium iodide stain. SYTO 9 stains both live and dead bacteria, whereas propidium iodide penetrates only bacteria having damaged membranes. When used together, propidium iodide reduces SYTO 9 fluorescence in dead bacteria with damaged membranes resulting in red fluorescent cells, whereas the live bacteria will fluoresce green [62]. 

### 7.3. Molecular Techniques

Hybridization probes are nucleic acids (DNA/RNA) which have been chemically or radioactively labeled and are used to detect complementary target DNA/RNA. Specific amplification of DNA targets in bulk DNA extracts from environmental and clinical samples permits detection of specific organisms or groups of related organisms without the need to cultivate them, provided the appropriate unique primers are used [63]. These procedures do not discriminate between culturable and nonculturable forms of the target organisms. Due to the failure of distinguishing between dead or live cells by DNA-based methods, RNA-based methods are a more valuable estimate of gene expression and/or cell viability under different conditions [64]. This technique is more able to discriminate between culturable and nonculturable forms of an organism. Furthermore, reverse transcriptase PCR (RT-PCR) can distinguish between live and dead cells. This is possible because it is an mRNA-based method and mRNA is short-lived (half-life less than 1 minute). Messenger RNA is only present in metabolically active cells and not found in nature after cell death. RT PCR can detect nonculturable but active or live cells [65].

Even though traditional culture methods fail to detect the presence of specific VBNC in a sample, the presence of these microbes can be demonstrated using some of the molecular techniques described. More specifically, oligonucleotide probes of l8–20 nucleotides are proving most useful because they hybridize rapidly to specific DNA sequences of target organisms. These gene probes can reveal closely related organisms or organisms with similar functional capabilities. Additional molecular techniques are then required to fully identify any VNBC detected [66]. The detection of VBNC cells directly from the environmental samples can also be achieved using different types of blotting such as colony blot, slot blot, dot blot, and southern blot. The principle of blotting is the use of radio- or nonradioactive or fluorescence labeled probe. Fluorescent in situ Hybridization (FISH) is an alternative format for hybridization probes in which fluorescence labeled DNA or RNA probes are hybridized with target nucleic acids in whole, permeabilized cells [17]. The application of this method to the detection of single microbial cells by using rRNA-targeted probes in combination with epifluorescent microscopy has been developed. This is done through selective targeting of regions of rRNA, which consist of conserved and variable nucleotide regions. By choosing the appropriate rRNA probe sequence, FISH can be used to detect all bacterial cells (a universal probe) or a single population of cells (a strain-specific probe) of VBNC. It has lower sensitivity and cannot distinguish live and dead cells [63].

## 8. Conclusion

From the above discussions, it is evident that a number of nonspore-forming human pathogenic bacteria can enter the VBNC state with maintained cellular structure and biology and persistent gene expression but remain nonculturable by traditional cultural techniques. They can survive and revert to culturable conditions when provided with appropriate conditions, hence, being a significant threat to public health and food safety. Further research is needed to elucidate mechanisms leading to the VBNC and the development of methodologies to confirm their existence.

## Figures and Tables

**Table 1:** Fluorescent dyes used for detection of VBNC bacteria.

Dye	Mechanism	Reaction
Acridine orange	The staining response depends on the ratio of DNA to protein in the cells	Actively reproducing cells appear green but slow-growing or nonreproducing cells at time of staining appear orange

Di-amino-phenyl-indole (DAPI)	Differential staining	Living cells look green under fluorescent microscope

Indophenyl-nitrophenyl-phenyl tetrazolium chloride (INT)	INT reacts with dehydrogenase enzyme to produce formazone and red color, thus living cells appear red	Living cells appear red.

Fluorescein isothiocyanate (FITC)	Enzyme activity in living cell	FITC stains living cells violet or blue
